# Game Animal Density, Climate, and Tick-Borne Encephalitis in Finland, 2007–2017

**DOI:** 10.3201/eid2612.191282

**Published:** 2020-12

**Authors:** Timothée Dub, Jukka Ollgren, Sari Huusko, Ruut Uusitalo, Mika Siljander, Olli Vapalahti, Jussi Sane

**Affiliations:** European Programme for Intervention Epidemiology Training, European Centre for Disease Prevention and Control, Stockholm, Sweden (T. Dub);; Finnish institute for Health and Welfare, Helsinki, Finland (T. Dub, J. Ollgren, S. Huusko, J. Sane);; University of Helsinki, Helsinki (R. Uusitalo, M. Siljander, O. Vapalahti);; Helsinki University Hospital, Helsinki (O. Vapalahti)

**Keywords:** animal population density, arboviruses, climate, deer, encephalitis, Finland, flaviviruses, foxes, game animals, hares, infectious diseases, Ixodes ricinus, Ixodes persulcatus, moose, tick-borne diseases, time-series analyses, vectorborne diseases, viruses, zoonoses

## Abstract

Tick-borne encephalitis (TBE) is an endemic infection of public health importance in Finland. We investigated the effect of ecologic factors on 2007–2017 TBE trends. We obtained domestic TBE case data from the National Infectious Diseases Register, weather data from the US National Oceanic and Atmospheric Administration, and data from the Natural Resources Institute in Finland on mammals killed by hunters yearly in game management areas. We performed a mixed-effects time-series analysis with time lags on weather and animal parameters, adding a random effect to game management areas. During 2007–2017, a total of 395/460 (86%) domestic TBE cases were reported with known place of exposure and date of sampling. Overall, TBE incidence increased yearly by 15%. After adjusting for the density of other animals and minimum temperatures, we found thatTBE incidence was positively associated with white-tailed deer density. Variation in host animal density should be considered when assessing TBE risks and designing interventions.

Tick-borne encephalitis (TBE) is an endemic vectorborne infectious disease of public health importance in Finland. It is caused by tick-borne encephalitis virus (TBEV), a member of the *Flavivirus* genus of the *Flaviviridae* family. TBEV has 5 subtypes: European and Siberian subtypes, whose presence in Finland has been established (*1**,**2*); recently described Himalayan subtype; Far Eastern subtype (*3*); and Baikalian subtype (*4*). TBEV is most often transmitted through the bite of *Ixodes ricinus* or *I. persulcatus* ticks, 2 species found in Finland that can carry both the European and Siberian TBEV subtypes (*5**–**7*). TBEV may also be transmitted through the consumption of unpasteurized dairy products from infected livestock (*8**,**9*). 

Most TBEV infections are asymptomatic (*10*). For clinical infections, the infectious course will differ depending on the TBEV subtype. The European subtype is typically responsible for a biphasic course of the disease: a short incubation period leads to a viremic phase associated with influenza-like symptoms, followed by an asymptomatic interval before onset of acute viral meningoencephalitis. Residual sequelae are reported in up to 50% of patients with European subtype TBE; the case fatality rate (CFR) is <2% (*10**,**11*). The Siberian subtype is associated with direct neurologic signs including focal encephalitis or meningitis in most symptomatic cases and complete recovery occurring in 80% patients; CFR approaches 2% (*10*). Effective TBE vaccines based on purified, formalin-inactivated TBEV are available, but several doses and boosters are required to acquire and maintain immunity.

In Europe, several thousand TBE cases are reported yearly, with the highest incidences in the Baltic countries (*12**,**13*). In Finland, TBE cases are reported from relatively restricted areas, mostly around the archipelago and the coast (*14*). During 1995–2013, the average annual number of cases was 25, ranging from 5 to 43 cases per year (*14*), but the incidence has increased over the past 5 years with the development of new TBE foci (*15*). 

Several seasonal or environmental factors, such as temperature (*16**,**17*) and humidity (*18*), along with the number of animal hosts for ticks feeding (*19*), have been shown to affect tick life cycles and activity, which in turn have been associated with transmission of tick-borne infections, such as Lyme disease (*18**,**20**,**21*) and TBE (*21**–**24*). Our aim was to assess the effects of environmental factors, game animal density, and temperatures on TBE emergence and distribution in Finland and to use these findings to inform risk assessment and prevention strategies. 

## Methods 

### Epidemiologic Data 

In Finland, TBE is reportable to the National Infectious Diseases Register (NIDR), maintained by the Finnish Institute for Health and Welfare (Terveyden ja hyvinvoinnin laitos [THL]). An acute laboratory-confirmed TBE case is defined as one in which a patient without a disease-specific medical history (e.g., no previous TBEV exposure) has coherent central nervous system symptomatology, such as meningitis, meningoencephalitis, or encephalomyelitis, and TBEV-specific IgM and IgG detected in either cerebrospinal fluid or serum. Two clinical laboratories in Finland perform TBE diagnostics and report results electronically to the NIDR. Each notification includes the specimen date and the patient’s unique national identity code, date of birth, sex, and place of residence. 

Since 2007, the Finnish Institute for Health and Welfare has enhanced TBE surveillance in place; because of these additional data, we were able to examine medical records and interview patients to determine the most likely places of exposure (*25*). From these sources, we extracted data on the number of TBE cases for 2007–2017. To account for a median TBE incubation period of »7–14 (range 4–28) days (*10*), suspected date of exposure was calculated as 2 weeks before date of symptom onset or 3 weeks before date of sampling if date of onset was unknown. 

### Weather and Game Animal Density Data

We retrieved temperature data from daily weather reports from multiple meteorologic stations in Finland using the United States National Oceanographic and Atmospheric Administration (NOAA) Climate Data Online open-access platform (https://www.ncdc.noaa.gov/cdo-web) (*26*). Including geographic coordinates for each station, we used QGIS 2.14.20-Essen version software (QGIS, https://qgis.org/en/site) to assign each station to its respective game management area and used Stata version 15 statistical software (StataCorp, https://www.stata.com) to calculate weather data for each game management area (n = 16). We used the difference between reported minimum and maximum daily temperatures to determine temperature variation, then calculated monthly mean values for minimum, maximum, average, and variation for each weather station. We used these data to calculate mean temperature values for the game management areas. 

We used mean daily temperature values from each game management area to calculate 2 other weather parameters that affect tick populations. We used the proportion of days in a month with a mean temperature >5°C because ticks are commonly encountered in the northern regions of Europe at that temperature level (*27**,**28*). We used monthly mean temperature surplus (mean temperature in degrees Celsius above 9°C (or 0, if <9°C) because it has been observed that *Ixodes ricinus* larval activity and development occurs at that temperature (*29*). 

For the spatial unit in our study, we used game management areas as defined by the Natural Resources Institute Finland. We obtained data for 2006–2017 on the number of animals killed by hunters in game management areas for moose (*Alces alces*), fallow deer (*Dama dama*), roe deer (*Capreolus capreolus*), white-tailed deer (*Odocoileus virginianus*), European hare (*Lepus europaeus*), mountain hare (*Lepus timidus*), and red fox (*Vulpes vulpes*) from the statistical services portal of the Natural Resources Institute Finland (Luonnonvarakeskus [Luke]) (*30*). We used these data on the numbers of animals killed by hunters as a proxy for actual animal density because they have been shown to be strongly correlated (*31**,**32*). To improve readability of our results, we divided the number of animals killed by hunters per game management areas by 100 (e.g., 5,500 animals = 5.5) and used this number in our models. We used a 1-year lag to estimate the level of TBE incidence based its demonstrated correlation with calculated animal density (*22*). 

### Statistical Analysis 

We calculated the monthly number of cases and temperature values for each game management area, then modeled annual TBE incidence in 2007–2017 at a national level and by game management area using negative binomial regressions, a type of generalized linear model used to model overdispersed count data. As our core model for this time-series analysis, we fit a mixed-effects negative binomial regression model with the monthly numbers of TBE cases reported from each game management area as an outcome and year-month unit (2010m1, 2010m2, etc.), adjusted for a 12-month periodicity as explanatory variables, with a random effect on game management areas to account for regional variability. 

For calculations, all temperature variables were used with a 1-month lag period, assuming that ticks would not become fully active again from the beginning of periods with optimal life cycle temperature ranges. By adding the variables to the core model one at a time, we determined the model that showed the best Akaike information criterion/Bayesian information criterion combination. The climatic predictor that offered the best fit was considered the most informative predictor, and we then used it as an adjustment variable when modeling the effect of the number of animals killed on TBE incidence for each game management area. We then conducted single-variable analyses of all animal density parameters per 100 units, with a 1-year lag. 

Finally, we used stepwise backward selection to develop a final multiple-variable analysis model to determine the effect of animal density on TBE incidence in a multilevel mixed-effects negative binomial regression with a random effect on game management areas. We assessed the distribution of Pearson and Deviance residuals and looked for autocorrelation of residuals. Statistical significance was considered at a 5% level. We used Stata to perform modelling; results were displayed with incidence ratios (IR) to 2 decimal places with 95% CIs and, when relevant, negative binomial regression coefficients before exponentiation to 3 decimal places with 95% CIs. 

## Results 

### Epidemiology of TBE Cases

**Figure 1:**
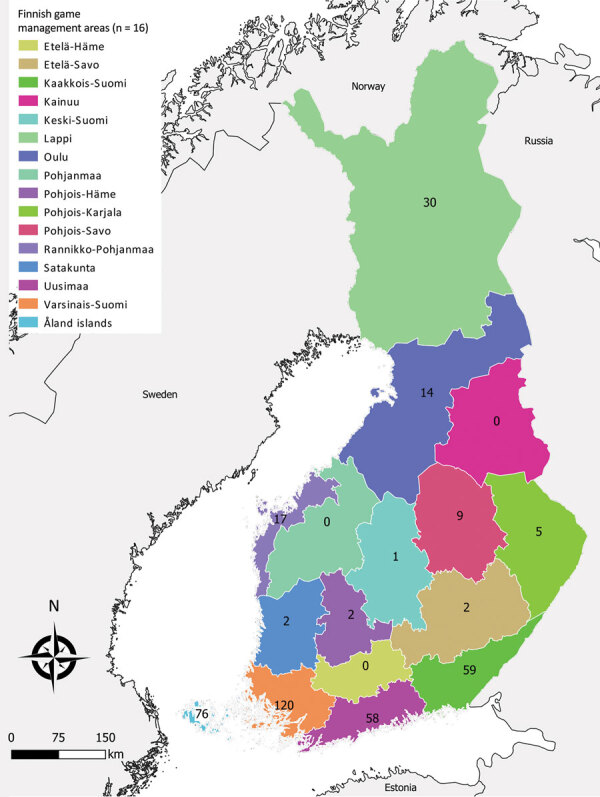
Total number of tick-borne encephalitis cases reported by game management area, Finland 2007–2017.

During 2007–2017, a total of 488 cases were reported to NIDR, including 28 (6%) with reported exposure in a foreign country (Estonia, 18; Sweden, 5; Russia, 2; other, 3) and 65 cases (13%) without known place of exposure or date of sampling. We included in the analysis the remaining 395 (81%) cases of domestic TBE reported to NIDR. Median yearly number of cases was 28 (interquartile range [IQR] 20–50). Over the study period, the median number of cases was 7 per management area, ranging from 0 in several central game management areas to 120 cases in southern Finland (Varsinais-Suomi) (Figure 1). Overall, nationwide domestic TBE incidence significantly increased by 15% (IR 1.15; 95% CI 1.10–1.20) yearly, with regional variation (Table 1). 

**Table 1:** Yearly tick-borne encephalitis incidence increase nationwide and by game management area, Finland 2007–2017

Game management area	Median annual no. cases (IQR)†	Annual no. cases, range	IR (95% CI)		p value	Yearly trend, %
Åland islands	7 (2–11)	1–14	0.97 (0.85–1.10)		0.60	–3%
Etelä-Häme	None reported	0–0				
Etelä-Savo	0 (0–0)	0–1	1.17 (0.73–1.87)		0.51	+17%
Kaakkois-Suomi	5 (2–6)	1–15	1.28 (1.04–1.32)		<0.01	+28%
Kainuu	None reported	0–0				
Keski-Suomi	0 (0–0)	0–1	1.46 (0.61–3.52)		0.40	+46%
Lappi	2 (1–3)	0–9	1.23 (1.08–1.40)		<0.01	+23%
Oulu	0 (0–3)	0–4	1.50 (1.18–1.92)		0.001	+50%
Pohjanmaa	None reported	0–0				
Pohjois-Häme	0 (0–0)	0–2	1.34 (0.31–5.80)		0.70	+34%
Pohjois-Karjala	0 (0–1)	0–1	1.21 (0.89–1.65)		0.22	+21%
Pohjois-Savo	0 (0–2)	0–3	1.21 (0.96–1.53)		0.11	+21%
Rannikko-Pohjanmaa	1 (0–2)	0–6	0.80 (0.65–1.00)		0.05	–20%
Satakunta	0 (0–0)	0–1	3.00 (0.60–14.87)		0.18	+200%
Uusimaa	4 (1–9)	0–16	1.39 (1.25–1.54)		0.001	+39%
Varsinais-Suomi	7 (4–17)	3–28	1.23 (1.15–1.31)		0.001	+23%
Finland	28 (20–50)	17–73	1.15 (1.10–1.20)		0.001	+15%

*IQR, interquartile range; IR, incidence ratio. †None reported indicates that 0 cases were reported in that management area over the entire study period.

### Association of TBE Incidence with Game Animal Density and Weather-Based Parameters 

We used data from 174 different weather stations in total; the number of stations assigned to each game management area ranged from 3 in the Åland Islands to 47 in the Lappi (Lapland) game management area (Appendix Table 1). The median number of daily reports available per station per month was 30 (IQR 30–31). The number of animals killed by hunters by area was available on a yearly level for all game management areas except for the Åland Islands because the Natural Resources Institute Finland did not collect data for this management area. 

Our core mixed-effects negative binomial regression showed a 1% monthly increase (IR 1.01 [1.01–1.01]; coefficient 0.011 [0.008–0.149]) and a significant 12-month periodicity (p<0.001). None of the temperature variables showed a significant association with monthly TBE incidence (Table 2). The lowest Akaike information criterion/Bayesian information criterion combination was seen for monthly average minimum temperature; therefore, we adjusted our modeling of hunting data for this weather parameter. 

**Table 2:** Single-variable modeling of weather parameters’ influence on tick-borne encephalitis incidence, Finland, 2007–2017

Weather parameters†	Coefficient (95% CI of coefficient)	IR (95% CI)	p value	AIC	BIC
Monthly average minimum temperature	–0.069 (–0.145 to 0.08)	0.93 (0.87–1.01)	0.08	1,263.0	1,302.6
Monthly average mean temperature	–0.070 (–0.149 to 0.008)	0.93 (0.86–1.01)	0.08	1,263.1	1,302.6
Monthly average maximum temperature	–0.056 (–0.126 to 0.013)	0.95 (0.88–1.01)	0.11	1,263.6	1,303.1
Monthly average of daily temperature variation	0.000 (–0.103 to 0.104)	1.00 (0.90–1.11)	0.99	1,266.1	1,305.6
Proportion of days in a month with a mean temperature >5°C	–0.718 (–1.702 to 0.265)	0.49 (0.18–1.30)	0.15	1,264.1	1,303.6
Monthly average of mean temperature surplus‡	–0.057 (–0.135 to 0.021)	0.94 (0.87–1.02)	0.15	1,264.0	1,303.6

*AIC, Akaike information criterion; BIC, Bayesian information criterion; IR, incidence ratio. †Adjusted for trend over time and 12 mo periodicity. ‡Temperature surplus: mean temperature minus 9°C if ≥9°C, otherwise 0.

Because data on mammals killed by hunters were not available for the Åland Islands game management area, our analysis was restricted to 15 of the 16 game management areas (Appendix Table 2). Our single hunting data variable analysis, using a mixed-effects negative binomial regression, adjusted for year-month time unit, average minimum temperature, periodicity, and a random effect on game management areas, showed no significant results (Appendix Table 3). The numbers of moose, roe deer, and fallow deer were negatively associated with TBE incidence trends; numbers of fox, white-tailed deer, European hare, and mountain hare were positively associated with TBE incidence trends, although not significantly. 

Using a stepwise approach for our analysis of the effect of several animal densities adjusted for average monthly minimum temperature, we obtained a model containing the numbers of moose, roe deer, white-tailed deer, mountain hare, and fox killed by hunters (Appendix Table 4). We found that TBE incidence was positively associated with the number of white-tailed deer killed by hunters (IR 1.04 [1.01–1.07]; coefficient 0.037 [0.009–0.064]), but it was significantly negatively associated with the number of roe deer (IR = 0.94 [0.88–1.00]; coefficient −0.067 [–0.131 to −0.003]). Other animal densities yielded no significant results (Table 3); Pearson and deviance residuals were normally distributed around 0, and residuals only showed autocorrelation in 2 game management areas out of 14 included in the model.

**Table 3:** Ecologic parameters associated with tick-borne encephalitis incidence, Finland, 2007–2017*

Species	Coefficient (95% CI of coefficient)	IR (95% CI)	p value	AIC	BIC
Moose (*Alces alces*)	–0.011 (–0.025 to 0.002)	0.99 (0.98–1.00)	0.11	1034.1	1101.1
Roe deer (*Capreolus capreolus)*	–0.067 (–0.131 to –0.003)	0.94 (0.88–1.00)	0.04		
White-tailed deer (*Odocoileus virginianus)*	0.037 (0.009–0.064)	1.04 (1.01–1.07)	0.01		
Mountain hare (*Lepus timidus)*	0.004 (–0.000 to 0.008)	1.00 (1.00–1.01)	0.08		
Red fox (*Vulpes vulpes*)	0.007 (–0.001 to 0.015)	1.01 (1.00–1.02)	0.09		
*Based on the yearly number of moose, roe deer, white-tailed deer, mountain hare, and red fox killed by hunters, adjusted for average minimum temperature with a 1-month lag, trend over time, and 12-month periodicity. AIC, Akaike information criterion of the multivariable time series model; BIC, Bayesian information criterion of the multivariable times series model; IR, incidence ratio.					

**Figure 2:**
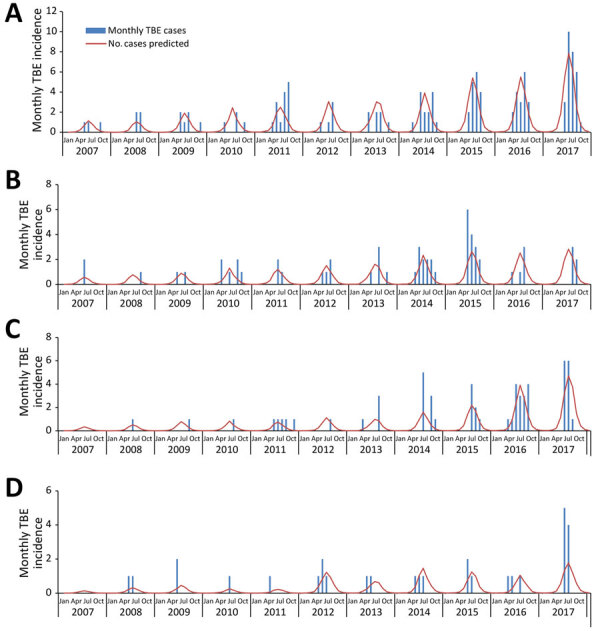
Actual and predicted number of TBE cases in 4 game management areas, Finland, 2007–2017. A) Varsinais-Suomi; B) Kaakkois-Suomi; C) Uusimaa; and D) Lappi. Number of tick-borne encephalitis cases is predicted by a mixed effects multivariable negative binomial model including number of moose, roe deer, white-tailed deer, mountain hare, and red fox killed by hunters adjusted for a 12-month periodicity, minimum temperature, and month, with a random effect on game management areas. TBE, tick-borne encephalitis

Figure 2 presents the actual monthly number of TBE cases and the model’s prediction for the 4 game management areas included the model that contributed the most to TBE incidence: Varsinais-Suomi, Kaakkois-Suomi, Uusimaa, and Lappi. These results show that our model failed to predict some incidence peaks; however, the yearly average difference between the actual number of cases and the model’s prediction was 0.04, ranging from −0.05 in Etela Hame to 0.19 in Uusimaa. 

## Discussion

During 2007–2017, TBE incidence in Finland increased yearly by 15%. Our analysis did not find a statistically significant association between temperature and TBE incidence. Multivariable analysis of the effect of several animal densities showed that TBE incidence was positively associated with the number of white-tailed deer, but an increase in the number of roe deer killed by hunters led to a decrease in TBE incidence. Our study provides further evidence on the importance of wildlife in the epidemiology of TBE. Blood meals are necessary for 3 different stages of tick development; large mammals such as deer can both serve as transmission hosts and provide blood meals to ticks, and their numbers can therefore have a strong influence on TBE rates (*22*). 

The results of our analysis of weather data were not consistent with those in many other studies on the effect of changes in temperature on TBE incidence (*22**,**33**,**34*). Regarding animal data, our results were partly in line with a 2017 study conducted in Sweden in which single-variable analysis with a 1-year lag showed that the number of fallow deer and moose killed by hunters were negatively associated with TBE incidence, but contrary to our findings, the number of roe deer had a positive effect on TBE increase (*22*). Roe deer abundance is a parameter that has previously been associated with TBE incidence in other TBE endemic areas. In northern Italy, it was shown that roe deer density was higher in areas where more TBE cases were detected (*23*); however, in Slovenia, when both red deer and roe deer density were studied, only red deer density showed a significant positive association with TBE incidence (*24*). Similarly, in the Czech Republic, in a multivariable model adjusted for forest and agriculture area, only the number of wild boars killed by hunters had a significant positive association with TBE incidence, whereas roe deer density was negatively, but nonsignificantly, associated with TBE incidence (*35*). However, the results of these studies cannot all be properly compared with the findings from our work due to different methodologies, fauna, the presence in Finland of 2 tick species (*Ixodes ricinus* and *I.* *persulcatus*) capable of transmitting TBEV, and differing temperature and environmental characteristics. For example, a study similar to ours was recently conducted in Sweden, a neighboring country with similar climate and fauna, but the species of deer studied differed (*22*). 

White-tailed deer are nonnative in Finland, introduced by the mid-20th century; the species’ numbers have grown from fewer than 10 to several hundred thousand (*36**,**37*). A geospatial modeling study using similar data recently showed that in Finland, the density of this animal was correlated with concurrent incidence rates of TBE (*38*), which is in line with our findings. These known ticks (*I. ricinus* and *I. persulcatus*) (*39*) and the TBE host (*40*) were also introduced in the Czech Republic (*41*), another TBE-endemic country; however, to our knowledge, their effect on incidence trends there has not been studied. 

As in any ecologic study, our results have to be interpreted with caution and should not be generalized to an individual level; some unmeasured characteristics might also differ between game management areas (*42*). In addition, we identified several limitations to our work. First, because we had to use average values for large geographic areas (median area size 19,185 km^2^ [IQR 15,826–21,589 km^2^]), the effect of temperatures on TBE incidence might have been diluted, which would explain why in our analysis, an increase in temperature was not linked to an incidence increase. This effect would also apply for animal density, which may vary within a game management area. Second, we were not able to use precipitation levels, a parameter with a known influence on tick lifecycle and activity, because data were not collected in a systematic manner throughout Finland. Third, because animal density per game management areas was not available, we used data on animals killed by hunters. Even though these data have been used by several researchers in similar studies on TBE (*22*) and Lyme disease, another tick-borne zoonotic disease (*20*), we cannot assume that it perfectly reflects animal density variations. The reported number of animals killed by hunters can also be affected by changes in hunting habits and wildlife population control regulations. Finally, the effect of density of other potential hosts of ticks and TBEV, such as smaller animals (e.g., rodents), could not be investigated because of lack of available data in Finland. The absence of data on precipitation levels and small rodent density might partially explain why our model failed to properly predict several sudden increases, such as in Varsinais-Suomi in 2017, Kaakkois-Suomi in 2015, or over the final years of the study period in the Uusimaa and Lappi game management areas (Figure 2). 

The effect of environmental factors, including climate change and host animal density variations, on vectorborne diseases is a growing concern in Finland. Therefore, over the coming years, the Finnish Institute for Health and Welfare will participate in a national consortium to quantify factors driving vectorborne diseases. The project will use modern analysis tools, empirical field studies, and predictive spatiotemporal modeling to provide information for intervention strategies integrating data on human disease incidence, dynamics of host communities, and vectors and environmental variables, including climate (*43*). We hope this project will lead to better knowledge about the extent and effects of climate change and milder temperatures and the influence of certain animal hosts on TBE show that incidence because it is growing in the European region (*12**,**13*). The findings of our study, especially that white-tailed deer density is associated with the incidence rates of TBE, show that variations in host animal density should be taken into account when assessing regional TBE risk, forecasting future trends, and designing interventions. Experimental studies on reducing or restricting the movement of deer populations (with fences) should assess whether such interventions can be effective to control tick populations and decrease TBE incidence. 

Appendix Additional information on variables affecting tick-borne encephalitis in Finland.
