# Supplementary material for: Game Animal Density, Climate, and Tick-Borne Encephalitis in Finland, 2007–2017
Source: Emerg Infect Dis. 2020 Dec;26(12):2899–906. doi: 10.3201/eid2612.191282 (PMC7706931; doi:10.3201/eid2612.191282)
Supplement: Appendix — Additional information on variables affecting tick-borne encephalitis in Finland. [file 19-1282-Techapp-s1.pdf]

Article DOI: https://doi.org/10.3201/eid2612.191292

Game Animal Density, Climate, and Tick-borne Encephalitis in Finland, 2007–2017

Appendix

Appendix Table 1. Size of game management areas, number of weather stations, and median monthly weather parameters by game management area, Finland 2007–2017

Game management areas	Area, km^2	No. of weather stations	Avg. minimum temp., °C (IQR)	Avg. mean temp., °C (IQR)	Avg. maximum temp., °C (IQR)	Avg. of daily temp. variation, °C (IQR)	Proportion of d per mo. with mean temp. >5°C (IQR)	Avg. of mean *temp. surplus, °C (IQR)
Åland islands	13,359.4	3	2.2 (–1.2 to 7.7)	5.6 (1.5–12.7)	9.2 (3.7–16.4)	7.2 (5.5–8.7)	0.6 (0.1–1.0)	0.1 (0.0–3.8)
Etelä-Häme	9,201.3	7	0.8 (–3.5 to 7.5	4.4 (–0.7 to 12.8)	8.8 (1.3–17.8)	8.4 (5.9–10.1)	0.4 (0.0–1.0)	0.1 (0.0–4.1)
Etelä-Savo	21,618.3	11	0.8 (–5.1 to 7.9)	4.0 (–2.0 to 12.7)	7.9 (0.3–17.3)	7.9 (6.2–9.6)	0.4 (0.0–1.0)	0.0 (0.0–4.0)
Kaakkois-Suomi	14,677.8	11	0.5 (–3.9 to 8.0)	4.5 (–1.2 to 13.0)	8.3 (1.0–17.8)	8.2 (6.1–10.0)	0.4 (0.0–1.0)	0.1 (0.0–4.2)
Kainuu	22,674.7	6	−1.6 (–7.9 to 5.7)	2.3 (–4.5 to 11.2)	5.4 (–1.5 to 15.7)	8.6 (6.7–10.1)	0.2 (0.0–1.0)	0.0 (0.0–2.7)
Keski-Suomi	18,432.0	6	0.6 (–4.7 to 7.7)	3.7 (–1.7 to 12.3)	7.5 (0.5–16.8)	7.7 (5.8–9.6)	0.4 (0.0–1.0)	0.0 (0.0–3.5)
Lappi	100,300.9	47	−3.6 (–11.6 to 4.9)	0.1 (–6.8 to 9.0)	2.9 (–3.1 to 13.2)	8.5 (7.2–9.3)	0.0 (0.0–0.9)	0.0 (0.0–1.4)
Oulu	45,055.0	15	−1.7 (–7.3 to 5.7)	2.1 (–3.7 to 10.8)	5.4 (–0.8 to 15.3)	8.4 (6.6–9.8)	0.1 (0.0–1.0)	0.0 (0.0–2.6)
Pohjanmaa	21,326.6	9	−0.4 (–5.5 to 5.4)	3.5 (–1.7 to 12.1)	7.7 (0.6–17.0)	9.3 (6.7–11.2)	0.3 (0.0–1.0)	0.0 (0.0–3.4)
Pohjois-Häme	10,837.6	8	0.6 (–4.4 to 7.3)	4.1 (–1.2 to 12.5)	8.2 (1.0–17.4)	8.4 (6.1–10.2)	0.4 (0.0–1.0)	0.1 (0.0– 3.7)
Pohjois-Karjala	21,578.8	9	−0.7 (–6.3 to 6.7)	3.0 (–3.2 to12.0)	6.6 (–0.6 to 16.7)	8.2 (6.2–10.0)	0.2 (0.0–1.0)	0.0 (0.0– 3.6)
Pohjois-Savo	19,938.0	8	−0.2 (–6.0 to 7.6)	3.3 (–2.8 to 12.5)	7.0 (–0.2 to 17.0)	7.6 (6.0– 9.2)	0.3 (0.0–1.0)	0.0 (0.0– 3.6)
Rannikko-Pohjanmaa	18,114.8	7	1.3 (–3.8 to 7.5)	3.7 (–0.7 to 12.1)	7.3 (1.5–15.8)	7.4 (5.5–8.6)	0.4 (0.0–1.0)	0.0 (0.0–3.3)
Satakunta	16,208.9	7	0.9 (–3.6 to 7.4)	4.4 (–0.6 to 12.6)	8.5 (1.7–17.2)	8.3 (6.0–10.0)	0.4 (0.0–1.0)	0.0 (0.0–3.6)
Uusimaa	16,867.2	13	1.8 (–2.4 to 8.9)	5.3 (0.0–13.2)	8.7 (2.3–17.4)	7.6 (5.6–9.3)	0.6 (0.0–1.0)	0.3 (0.0–4.2)
Varsinais-Suomi	20,624.6	7	1.2 (–2.9 to 7.7)	4.8 (0.1–12.8)	9.3 (2.4–17.4)	8.4 (6.0–10.3)	0.4 (0.0–1.0)	0.2 (0.0–4.0)

avg, average; no, number; temp, temperature

*Temperature surplus: mean temperature in degrees Celsius above 9°C or 0, if <9°C

Appendix Table 2. Yearly median (Interquartile range) number of animals hunted per game management areas per 100 units, with a one-year lag, Finland 2007–2017

Game management areas	Moose (*Alces alce*)	Fallow deer (*Dama dama*)	Roe deer (*Capreolus capreolus*)	White-tailed deer (*Odocoileus virginianus*)	European hare (*Lepus europeaus*)	Mountain hare (*Lepus timidus*)	Red fox (*Vulpes vulpes*)
Åland islands	NA	NA	NA	NA	NA	NA	NA
Etelä-Häme	13.9 (11.4–15.3)	0.0 (0.0 – 0.1)	1.4 (1.3 – 2.1)	45.9 (33.7–48.9)	62.0 (54.0 - 71.0)	23.0 (21.0 – 35.0)	23.0 (18.0 – 29.0)
Etelä-Savo	35.8 (32.6–41.6)	0.0 (0.0–0.0)	0.4 (0.3–0.5)	0.5 (0.4–0.7)	31.0 (23.0–44.0)	114.0 (82.0–145.0)	20.0 (15.0–43.0)
Kaakkois-Suomi	23.1 (17.8–24.7)	0.0 (0.0–0.0)	0.6 (0.5–1.0)	0.8 (0.6–0.9)	44.0 (37.0–53.0)	58.0 (46.0–73.0)	25.0 (19.0–34.0)
Kainuu	37.0 (23.0–63.7)	0.0 (0.0–0.0)	0.1 (0.1–0.2)	0.0 (0.0–0.0)	8.0 (5.0–12.0)	185.0 (144.0–200.0)	20.0 (15.0–23.0)
Keski-Suomi	38.9 (32.7–46.5)	0.0 (0.0–0.0)	0.7 (0.5–0.8)	1.0 (0.8–1.2)	35.0 (29.0–42.0)	141.0 (101.0–172.0)	23.0 (20.0–35.0)
Lappi	100.4 (60.1–113.6)	0.0 (0.0–0.0)	0.5 (0.4–0.6)	0.0 (0.0–0.0)	0.0 (0.0–1.0)	171.0 (126.0–209.0)	92.0 (63.0–110.0)
Oulu	92.8 (56.2–123.2)	0.0 (0.0–0.0)	2.6 (1.9–2.9)	0.0 (0.0–0.0)	55.0 (40.0–84.0)	267.0 (242.0–325.0)	61.0 (45.0–83.0)
Pohjanmaa	37.8 (35.1–48.1)	0.0 (0.0–0.0)	3.7 (3.0–5.2)	2.1 (1.3–3.0)	117.0 (88.0–145.0)	84.0 (63.0–105.0)	53.0 (41.0–70.0)
Pohjois-Häme	17.5 (17.0–19.9)	0.0 (0.0–0.0)	1.4 (1.1–1.8)	25.8 (21.5–27.6)	34.0 (30.0–44.0)	37.0 (30.0–51.0)	18.0 (16.0–25.0)
Pohjois-Karjala	20.4 (19.8–23.4)	0.0 (0.0–0.0)	0.2 (0.2–0.4)	0.0 (0.0–0.0)	42.0 (38.0 - 48.0)	263.0 (205.0–287.0)	14.0 (11.0–22.0)
Pohjois-Savo	33.6 (29.6–38.8)	0.0 (0.0–0.0)	0.4 (0.2–0.5)	0.0 (0.0–0.1)	72.0 (44.0–90.0)	343.0 (239.0–441.0)	19.0 (11.0–22.0)
Rannikko-Pohjanmaa	23.1 (18.3–28.1)	0.0 (0.0–0.0)	3.1 (2.4–3.6)	3.0 (2.1–4.2)	15.0 (11.0–18.0)	24.0 (17.0–31.0)	17.0 (12.0–25.0)
Satakunta	22.5 (21.4–25.0)	0.1 (0.1–0.1)	5.1 (4.6–5.6)	48.6 (42.3–50.1)	51.0 (46.0–57.0)	21.0 (16.0–28.0)	36.0 (33.0–53.0)
Uusimaa	24.1 (18.7–26.7)	0.5 (0.4–1.1)	4.0 (3.3–5.7)	36.9 (32.3–41.1)	69.0 (61.0–95.0)	25.0 (20.0–38.0)	28.0 (18.0–48.0)
Varsinais-Suomi	18.5 (16.2–20.4)	0.2 (0.1–0.3)	12.8 (9.8–15.9)	82.4 (77.6–90.2)	59.0 (52.0–66.0)	12.0 (11.0–16.0)	44.0 (34.0–61.0)

NA, not available

Appendix Table 3. Single variable analysis of yearly number of animals killed by hunters per 100 units* associated with tick-borne encephalitis incidence, Finland, 2007–2017

Animal (genus and species)	Coefficient	95%CI of coefficient	IR	95% CI of IR	p-value	AIC	BIC
Moose (*Alces alces*)	–0.009	–0.022 to 0.004	0.99	0.98–1.00	0.19	1038.3	1083.0
Fallow deer (*Dama dama*)	–1.018	–2.242 to 0.206	0.36	0.11–1.23	0.10	1037.3	1082.0
Roe deer (*Capreolus capreolus*)	–0.002	–0.045 to 0.041	1.00	0.96–1.04	0.94	1040.1	1084.8
White-tailed deer (*Odocoileus virginianus*)	0.014	–0.005 to 0.033	1.01	0.99–1.03	0.16	1038.2	1082.9
European hare (*Lepus europeaus*)	0.001	–0.009 to 0.011	1.00	0.99–1.01	0.83	1040.1	1084.8
Mountain hare (*Lepus timidus*)	0.004	–0.000 to 0.008	1.00	1.00–1.01	0.05	1036.6	1081.3
Red fox (*Vulpes vulpes*)	0.007	–0.011 to 0.014	1.01	0.99–1.01	0.10	1037.4	1082.1

AIC, Akaike Information Criterion; BIC, Bayesian Information Criterion; CI, confidence interval; IR, incidence ratio
*Adjusted for average minimum temperature, trend over time and 12 mo periodicity

Appendix Table 4. Construction of the multivariable model of yearly number of animals killed by hunters per 100 units* associated with tick-borne encephalitis incidence, Finland, 2007–2017

Model	Animal (genus and species)	Coefficient	95% CI of coefficient	p-value	AIC	BIC
7-animal model	Moose (*Alces alces*)	−0.012	–0.026 to 0.001	0.08	1035.5	1113.7
	Fallow deer (*Dama dama*)	−0.991	–2.265 to 0.283	0.13		
	Roe deer (*Capreolus capreolus*)	−0.067	–0.131 to −0.002	0.04		
	White-tailed deer (*Odocoileus virginianus*)	0.037	0.009–0.065	0.01		
	European hare (*Lepus europeaus*)	−0.003	–0.015 to 0.008	0.56		
	Mountain hare (*Lepus timidus*)	0.003	–0.001 to 0.008	0.12		
	Red fox (*Vulpes vulpes*)	0.007	–0.001 to 0.008	0.10		
6-animal model	Moose (*Alces alces*)	−0.012	–0.025 to 0.002	0.09	1033.9	1106.4
	Fallow deer (*Dama dama*)	−0.930	–2.19 to 0.332	0.15		
	Roe deer (*Capreolus capreolus*)	−0.66	–0.130 to −0.001	0.05		
	White-tailed deer (*Odocoileus virginianus*)	0.037	0.009–0.065	0.01		
	Mountain hare (*Lepus timidus*)	0.003	–0.001 to 0.008	0.12		
	Red fox (*Vulpes vulpes*)	0.007	–0.002 to 0.015	0.12		
5-animal model (final model)	Moose (*Alces alces*)	−0.011	–0.025 to 0.002	0.11	1034.1	1101.1
	Roe deer (*Capreolus capreolus*)	−0.067	–0.131 to −0.003	0.04		
	White-tailed deer (*Odocoileus virginianus*)	0.037	0.009–0.064	0.01		
	Mountain hare (*Lepus timidus*)	0.004	–0.000 to 0.008	0.08		
	Red fox (*Vulpes vulpes*)	0.007	–0.001 to 0.015	0.09		

AIC, Akaike Information Criterion; BIC, Bayesian Information Criterion; CI, confidence interval
*Adjusted for average minimum temperature, trend over time and 12 mo periodicity
